# How Does Vaccination against SARS-CoV-2 Affect Hospitalized Patients with COVID-19?

**DOI:** 10.3390/jcm11133905

**Published:** 2022-07-05

**Authors:** Paloma Moreno-Nunez, Aurora Bueno-Cavanillas, Diego San Jose-Saras, Jorge Vicente-Guijarro, Abelardo Claudio Fernández Chávez, Jesús María Aranaz-Andrés, on behalf of Health Outcomes Research Group of the Instituto Ramón y Cajal de Investigación Sanitaria (IRYCIS)

**Affiliations:** 1 Department of Preventive Medicine and Public Health, Hospital Universitario Ramón y Cajal, 28034 Madrid, Spain; pmorenon@salud.madrid.org (P.M.-N.); jorge.vicente@salud.madrid.org (J.V.-G.); abelardoclaudio.fernandez@salud.madrid.org (A.C.F.C.); jesusmaria.aranaz@salud.madrid.org (J.M.A.-A.); 2 Instituto Ramón y Cajal de Investigación Sanitaria (IRYCIS), 28034 Madrid, Spain; 3 School of Medicine, Universidad Internacional de La Rioja, 26006 Logroño, La Rioja, Spain; 4 Department of Preventive Medicine and Public Health, Granada University, 18071 Granada, Spain; abueno@ugr.es; 5 Instituto de Investigación Biosanitaria de Granada (ibs.GRANADA), 18012 Granada, Spain; 6 Centro de Investigación Biomédica en Red de Epidemiología y Salud Pública (CIBERESP), Instituto de Salud Carlos III, 28029 Madrid, Spain; 7 Department of Medicine and Medical Specialities, School of Medicine, Alcalá University, 28034 Madrid, Spain

**Keywords:** COVID-19 vaccines, vaccine effectiveness, hospital mortality, critical care, hospitalisation

## Abstract

(1) Background: The development of effective COVID-19 vaccines has reduced the impact of COVID-19 on the general population. Our study aims to analyze how vaccination modifies the likelihood of death and length of stay in hospitalized patients with COVID-19; (2) Methods: A retrospective cohort study of 1927 hospitalized patients infected with COVID-19 was conducted. Information was gathered on vaccination status, hospitalization episode, and clinical profile of the patients. The effect of vaccination on mortality was analyzed using a multiple logistic regression model, and length of stay was analyzed using linear regression. The performance and fit of the models were evaluated; (3) Results: In hospitalized patients with COVID-19, the risk of dying during admission in vaccinated patients was half that of non-vaccinated (OR: 0.45; CI 95%: 0.25 to 0.84). In patients who were discharged due to improvement, the reduction in hospital stay in vaccinated patients was 3.17 days (CI 95%: 5.88 to 0.47); (4) Conclusions: Patients who, despite having been vaccinated, acquire the infection by SARS-CoV-2, have a significant reduction of the risk of death during admission and a reduction of hospital stay compared with unvaccinated patients.

## 1. Introduction

The SARS-CoV-2 pandemic is an unprecedented health emergency. In Spain, as of 31 January 2022, 9,961,253 cases of COVID-19 and 93,225 deaths had been confirmed, with the consequent overburdening of hospitals and intensive care units (ICU) [1]. Numerous studies have analyzed the determinants of severity and death due to COVID-19, constructing different predictive models based on patient or admission characteristics [2,3]. In the second half of 2021, the implementation of vaccination strategies changed the previous transmission behaviour and natural history of the disease [4,5,6]. The efficacy of vaccination in preventing symptomatic infection ranges from 60% to 95% depending on the type of vaccine [7]. Similarly, its effectiveness in preventing death, hospitalization, and intensive care unit (ICU) admission is increasingly well understood but varies according to the study population [8,9,10,11].

One group of particular interest, due to their high vulnerability, is that of hospitalized patients. Some papers have studied the characteristics of patients requiring hospital admission with COVID-19 despite prior vaccination, but evidence on the effect of immunization in this population remains scarce [11,12]. Given the decreasing efficacy of the vaccine over time, at least in the age groups most likely to require hospital admission [9,13], it is necessary to study whether prior vaccination against COVID-19 improves the clinical course and outcome of hospitalized patients.

The aim of this study was, based on a population of hospitalized patients with COVID-19, to analyze the effect of prior vaccination on the burden of disease measured by the length of hospital stay and the likelihood of ICU admission or death during the admission episode.

## 2. Materials and Methods

A retrospective cohort study was conducted on all patients with a confirmed microbiological diagnosis of COVID-19 admitted between 1 January and 31 July 2021 in a tertiary-level public hospital in the Community of Madrid (Spain). A microbiological diagnosis was conducted by a rapid antigen test or reverse transcription polymerase chain reaction, irrespective of the clinical picture. Of the patients who were admitted with COVID-19 more than once during the study period, only information relating to the first episode was included.

Subjects were classified into vaccinated and non-vaccinated. The former had to meet two criteria: (1) had received an appropriate vaccination schedule according to age, history of infection, and coexistence or not of high-risk clinical conditions [14] and (2) had received the last dose within a certain time frame (the time that each datasheet indicated was used to study the effectiveness of their vaccine) before the onset date of symptoms. The non-vaccinated group consisted of those individuals who had not received any doses of a vaccine at the onset date of symptoms. Subjects were excluded from the study if they had received an incomplete vaccination regimen or, having completed it, experienced symptoms before the time indicated in the technical guidelines had elapsed [7].

Age; gender; comorbidities present before admission, grouped by the Charlson index [15] (Supplementary Table S1); length of hospital stay; need for ICU stay; length of ICU stay; and admission outcome were obtained from the hospital admission service. The epidemiological surveillance system of the Preventive Medicine Service identified patients hospitalized with COVID-19 during the study period and provided information on the date of symptom onset in cases with a hospital début-onset of symptoms 48 h or more after admission. In cases of community début, the date of symptom onset was established, as observed in the course of the natural history of the disease, 5 days before the date of admission. The data relating to the type of vaccines administered, dates of administration, and belonging to groups with greater vulnerability were obtained from the Unified Vaccination Register of the Ministry of Health of the Community of Madrid.

The vaccines evaluated for effectiveness were those licensed in Spain at the time of the end of the participant recruitment period: Comirnaty (Pfizer-BioNTech; mRNA; 2 doses, 21 days between doses; immunity 7 days from second dose), Spikevax (Moderna; mRNA; 2 doses, 28 days between doses; immunity 14 days from second dose); Vaxzevira (AstraZeneca; Adenovirus ChAdOx1-S (recombinant); 2 doses; 8–12 weeks between doses; immunity 21 days from second dose); Janssen-Johnson & Johnson (Adenovirus Ad26.COV2-S (recombinant); 1 dose; immunity after 14 days) [7,16,17,18,19].

For categorical variables, absolute and relative frequencies were calculated, and for quantitative variables, mean and standard deviation or median and interquartile range (IQR) were calculated, according to their distribution. The distribution of the variables in the two groups of participants was studied with the chi-square test and Student’s *t*-test or Mann–Whitney U test, according to their nature and distribution.

Dependent variables were dichotomous (ICU admission and mortality) and continuous (length of hospital stay). Multiple, logistic, and linear regression models were used to identify and monitor confounding variables, respectively. To compare the length of hospital stay of vaccinated and non-vaccinated subjects, the model was constructed with subjects who did not die during admission.

In all cases, a backward modelling strategy was used, with an output *p*-value of 0.10. To correct for over-optimism of the logistic regression models and to assess their performance, bootstrap techniques were used; calibration was measured with the slope, while discriminatory ability was assessed with the area under the ROC curve (AUC). The goodness of fit of the linear regression model was measured by the R^2^ statistic.

The threshold for statistical significance was set at *p* = 0.05.

Statistical analysis was performed using Stata 16 statistical software (StataCorp LLC, College Station, TX, USA). 

The research was not funded. The study received approval from the Research Ethics Committee of the Hospital Ramón y Cajal on 22 December 2021 (reference: 328/21).

## 3. Results

**Figure 1:**
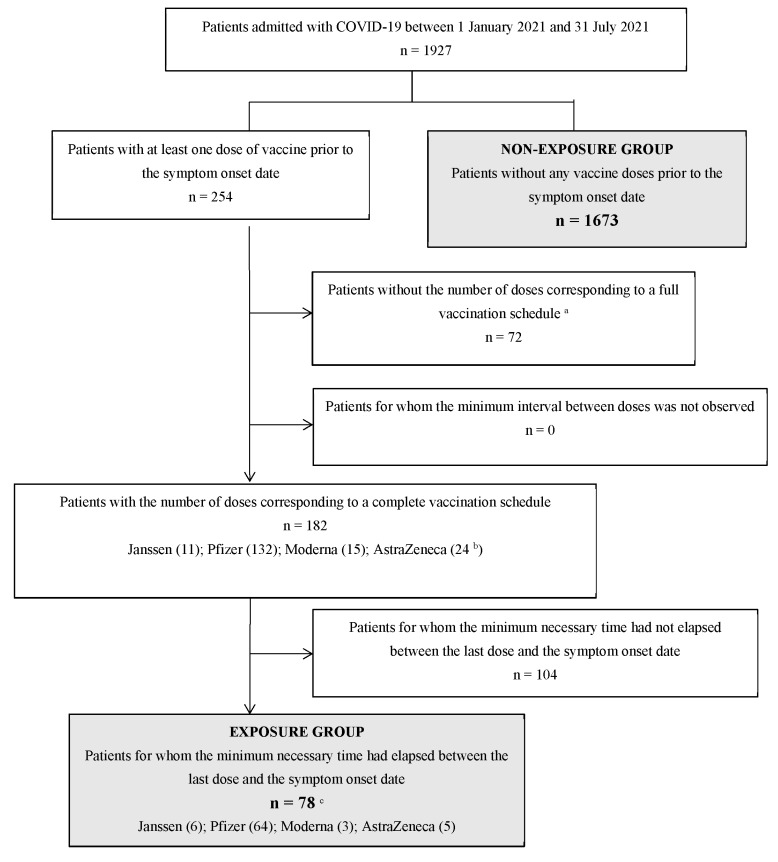
Diagram of participants’ selection and formation of the exposure groups. ^a^ No patient who had received a single dose of a vaccine other than the Janssen vaccine met the complete schedule criteria (history of past COVID, <65 years, and not belonging to groups with greater vulnerability). ^b^ One patient received a heterologous schedule: Vaxzevira (AstraZeneca) + Comirnaty (Pfizer/Biontech). The rest received homologous schedules. ^c^ All patients finally classified as vaccinated received homologous schedules; all of which were administered following the intervals between doses indicated in the datasheets. No patient received more than two doses.

During the study period, 1927 patients with confirmed COVID-19 were admitted (Figure 1). 1673 patients did not receive any doses of the vaccine, and 254 received at least one dose of the vaccine before the onset of symptoms. Of the 254 who had at least one dose of the vaccine, 72 were excluded because of receiving just one dose of a vaccine that required two, and 104 were excluded because they did not complete the time necessary to generate immunity. A total of 78 patients had an adequate vaccination schedule and had the minimum interval indicated in the corresponding technical guidelines having elapsed between the last dose received and the symptom onset date.

A total of 71 patients were vaccinated because of age criteria or because they were institutionalized in nursing homes, 2 were vaccinated because they suffered from a high-risk clinical condition, 2 because they were non-institutionalized dependent patients, 1 because of living in an institution other than a nursing home, and 1 because of being a nursing professional. The reason for vaccination of the remaining patient could not be identified. 

Neither age, Charlson index, length of hospital stay, nor length of ICU stay conformed to a normal distribution. The median age of the sample was 68 years (IQR 66–69) (Table 1). There were statistically significant differences between the median of the vaccinated people (86 years; Supplementary Figure S1) and non-vaccinated (67; *p* < 0.001) groups.

Although the median Charlson index was higher in the vaccinated participants than in the non-vaccinated group, the minimum and maximum Charlson index values in the vaccinated group were 0 and 9, respectively, while in the non-vaccinated group, they were 0 and 12.

The median hospital stay was 8 days in the total sample, with no difference between the vaccinated group (9 days) and the non-vaccinated group (8 days; *p* = 0.064).

Of the 1751 patients, 252 (14.39%) required admission to the ICU. The chi-square test found no statistically significant difference in the frequency of ICU admission between vaccinated (9; 11.54%) and non-vaccinated patients (243; 14.52%; *p* = 0.463). 

Table 1 shows that vaccinated patients were older and had more comorbidities, as well as a higher percentage of death. 

The median length of stay in ICU was 10 days in the total sample (IQR 5 to 17), being 5 in vaccinated people (IQR 1 to 12) and 10 in non-vaccinated people (IQR 5 to 17), although no statistically significant differences were found between the two groups (*p* = 0.054).

Table 2 shows the analysis of the variables associated with mortality. A crude analysis suggests an increased risk of mortality in vaccinated people, which is reversed when adjusting for age, previous comorbidity, and ICU admission. All these variables are associated with a significantly higher risk of in-hospital mortality.

The explanatory model that most accurately quantified the effect of vaccination on mortality, constructed from vaccination status, gender, ICU admission, age, and Charlson index found that a history of full vaccination reduced the risk of death by slightly more than half (OR: 0.45; CI 95%: 0.25 to 0.84) (Table 2).

The need for ICU care increased the risk of death the most, with those who died being five-times more likely to need critical care than not (OR: 4.88; CI 95%: 3.28 to 7.27). Gender was also a predictor of mortality and, in terms of OR, being male increased the risk of death by 78% (OR: 1.77; CI 95%: 1.31 to 2.41).

Age was a variable that was also associated with death, with each year’s increase adding 10% to the risk of death (OR: 1.09; CI 95%: 1.08 to 1.11). The Charlson index was also associated, increasing the risk by 20% for each one-point increase on the scale (OR: 1.20; CI 95%: 1.11 to 1.29). 

The goodness of fit of the model was studied by means of a calibration curve (Supplementary Figure S2), in which it was observed that the model performed optimally at low risks, noting a ratio of expected and observed events of 0.996 and a Slope of 0.97. The discrimination capacity measured by the area under the ROC curve was 0.83.

The explanatory model quantifying the effect of vaccination on the probability of ICU admission during admission, constructed from vaccination status, gender, age, and Charlson index, found no relation between vaccination status and ICU admission (OR: 1.00; CI 95%: 0.49 to 2.01) (Table 3).

Analysis of length of stay showed that, adjusting for the main variables, vaccination reduced hospital stay in these patients by 3.17 days (CI 95%: 5.88 to 0.47) (Table 4).

The condition that increased the length of stay the most was that of being male, which recorded an increase of 3.29 days (CI 95%: 1.95 to 4.64), followed by the Charlson index (1.53 days; CI 95%: 0.89 to 2.19). However, the R2 was 0.05, and the graphical analysis of the residuals showed that although the trend of the data was likely to be linear, it could not be assured that the assumption of equality of variances was met (Supplementary Figure S3).

## 4. Discussion

In subjects who, despite being properly vaccinated, were admitted to hospital with COVID-19, vaccination reduced the probability of death during admission by half. No difference was found in the risk of ICU admission, but hospital stays were shortened by 3 days. Taking into consideration that 78 vaccinated patients were monitored during the study period and that the cost of one day’s stay in our center without ICU admission is 340.98 €, vaccination resulted in a saving of 234 days of stay and 79,789.32 €.

It is worth noting that the explanatory model constructed has a considerably good predictive capacity for mortality, even though it was not designed for that purpose. This is probably because we were able to include in the research, in addition to age, the comorbidity of the patients, which are variables closely linked to a worse prognosis [20]. This, together with the sample size and the inclusion of all types of vaccines licensed in Spain are the main strengths of our work. However, its design implicitly assumes equal effectiveness of the different branded vaccines. It should be noted that their differences were reduced when measuring hard variables, such as mortality or ICU admission. A total of 82.05% of vaccinated patients had received Comirnaty (Pfizer/BioNTech); the figure rose to 85.90% if the classification was narrowed down to mRNA versus vectored virus vaccines; thus, heterogeneity in the vaccinated group was not excessively high. This, resulting in a limited sample size per vaccine type, prevented a more detailed analysis. This circumstance, together with others that could not be assessed, such as the causative variants of each infection, the history of SARS-CoV-2 infection, the clinical severity of the patient’s condition during admission, or the existence of criteria for admission to the ICU (the decision to admit the patient to the unit does not depend exclusively on the severity of that patient, but also on his or her chances of survival), provided residual confusion, which must be taken into consideration when interpreting the results. 

The fact that age, comorbidity factor, and mortality were higher in the vaccinated than non-vaccinated subjects may be explained by the structure of the vaccination campaign, which started on 27 December 2020 and prioritized immunization of older people and those with high-risk clinical conditions. By 31 July 2021, vaccination had been extended to everyone over 18 years of age and to individuals aged 12–18 years with high-risk clinical conditions, and the percentage of the total population of the Community of Madrid with a complete vaccination schedule was 54.08% [21]. 

The decrease in mortality observed in hospitalized patients is consistent with that described in the community, where mortality among non-vaccinated subjects aged 60–79 years, the median age range of the study participants, was 19.8-times higher than in vaccinated subjects [9]. The series studied is also consistent with the results of other authors regarding the worse prognosis associated with the male gender, both in relation to mortality and length of stay [22,23]. However, our results cannot support the protective effect of the vaccine on the likelihood of ICU admission [5], probably due to the small number of vaccinated subjects admitted to the ICU in our study and the uneven distribution of age and comorbidities in the exposure groups. 

Kissling et al. [24] and López Bernal et al. [25] studied the effect of the vaccine in those who experience symptoms before the time specified in the technical guideline as necessary for adequate protection to be generated. They found minor effects (14% for protection against infection if symptoms appeared between days 1 and 4 after vaccination and 32% if symptoms appeared between days 5 and 13), which did not always reach statistical significance. They attributed this to the fact that vaccinated subjects, due to post-vaccination influenza-like illness, are more frequently tested for SARS-CoV-2 than non-vaccinated subjects. On the other hand, subjects with symptoms suggestive of COVID-19 or recent contact with a sick person should postpone vaccination. Both situations bias the association against the null.

In our study, a limitation is that it was performed in one single center. Another one is the difference between vaccinated and non-vaccinated groups included in the study. Despite this, the benefit of vaccination is obvious. However, the results should be interpreted with caution, as with everything to do with this disease. Firstly, this is because of the still uncertain duration of immunity conferred by vaccination [26]. In our study, in no case did more than 6 months elapse between the date of the last vaccine dose and the date of hospital admission. On the other hand, it is necessary to maintain the level of epidemiological alertness for the early detection of new variants of the virus that could escape the protective immunity induced by the vaccine and thus modify the evolution of the pandemic [27,28].

Even so, the evidence of the protective effect of vaccination on mortality and length of stay in a population whose age and comorbidities make it more difficult to generate an adequate and durable response to vaccination underlines the importance of vaccination in reducing the burden of disease associated with the pandemic. At the same time, since the protective effect of vaccination is not universal, i.e., not all vaccinated people develop protective immunity, it is essential to remember that transmission control measures must persist as long as the virus continues to circulate.

The results obtained allow us to conclude that already in the first stage of the vaccination campaign, vaccinated patients who required hospital admission found that their risk of death was reduced by more than half. Those who survived the disease had shorter hospital stays, reducing the burden on the care units. These results are consistent with the findings by Stupica et al. in 2022, who found that vaccinated patients had half the risk of developing the severe disease and shorter stays [29]. However, this study shows that although vaccination against COVID-19 significantly reduced the risk of death, 20.5% of vaccinated patients died, so vaccination against COVID-19 should not be the only tool to mitigate the impact of the disease, and the development of specific pharmacological therapies is also necessary.

## Figures and Tables

**Table 1:** Description of the participants’ characteristics.

	Global		Non-Vaccinated		Vaccinated		*p*-Value
	(*n* = 1.751)		(*n* = 1.673; 95.55%)		(*n* = 78; 4.45%)		
**Age in years, median (IQR)**	68.04	54.02 to 81.28	67.34	53.67 to 80.01	86.20	76.37 to 91.58	<0.001
**Gender, *n* (%)**							
Women	746	42.60%	711	42.50%	35	47.87%	0.679
Male	1005	57.40%	962	57.50%	43	55.13%	
**Charlson Index, median (IQR)**	1	0 to 2	1	0 to 2	2	1 to 4	<0.001
**Hospital stay in days, median (IQR)**	8	4 to 15	8	4 to 15	9	6 to 15	0.065
**Admission to ICU, *n* (%)**							
No	1499	85.61%	1430	85.48%	69	88.46%	0.463
Yes	252	14.39%	243	14.52%	9	11.54%	
**ICU stay, median (IQR)**	10	5 to 17	10	5 to 17	5	1 to 12	0.054
**Outcome, *n* (%)**							
Improvement	1499	85.61%	1437	85.89%	62	79.49%	0.115
Death	252	14.39%	236	14.11%	16	20.51%	

IQR: interquartile range; ICU: intensive care unit.

**Table 2:** Mortality analysis.

	Crude Odds Ratio	CI 95%	Adjusted Odds Ratio ^a^	CI 95%
Fully vaccinated ^b^	1.57	0.89 to 2.77	0.45	0.25 to 0.84
Gender ^b^	1.24	0.95 to 1.64	1.77	1.31 to 2.41
Admission to ICU ^b^	2.07	1.49 to 2.87	4.88	3.28 to 7.27
Age (years)	1.08	1.07 to 1.09	1.09	1.08 to 1.11
Charlson	1.34	1.25 to 1.43	1.20	1.11 to 1.29
Length of hospital stay (days)	1.01	1.00 to 1.02	-	-
Length of stay in ICU (days)	1.00	0.98 to 1.02	-	-

^a^ OR of vaccination adjusted for gender, ICU admission, age, and Charlson index. ^b^ Reference values in categorical variables: 0: Not vaccinated; 0: Females; 0: No ICU stay.

**Table 3:** Explanatory model of ICU admission.

	Crude Odds Ratio	CI 95%	Adjusted Odds Ratio	CI 95%
Full vaccination ^a^	0.77	0.38 to 1.56	1.00 ^b^	0.49 to 2.01
Gender ^a^	1.62	1.22 to 2.14	1.42	1.07 to 1.87
Age (years)	0.98	0.98 to 1.00	0.98	0.97 to 0.99
Charlson	0.99	0.91 to 1.07	1.04	0.96 to 1.13
Constant	-	-	0.47	0.41 to 0.54

^a^ Reference values in categorical variables: 0: Non-vaccinated; 0: Females. ^b^ OR of vaccination adjusted for gender, age, and Charlson index.

**Table 4:** Linear regression model of hospital stay in patients discharged on the basis of improvement.

	Coefficient	CI 95%
Full vaccination ^a^	−3.17 ^b^	−5.88 to −0.47
Gender ^a^	3.29	1.95 to 4.64
Age (years)	0.05	0.02 to 0.09
Charlson	1.54	0.89 to 2.19
Constant	5.19	2.81 to 7.56

^a^ Reference values in categorical variables: 0: Non-vaccinated; 0: Females. ^b^ Coefficient of vaccination adjusted for gender, age, and Charlson index.

## Data Availability

The database can be made available upon request to the authors.

## Supplementary Materials

The following supporting information can be downloaded at https://www.mdpi.com/article/10.3390/jcm11133905/s1, Table S1: Charlson Index; Figure S1: Temporal distribution of receipt of the first dose of the vaccine according to the age group to which they belonged; Figure S2: Calibration plot of mortality model; Figure S3: Linear regression model residuals analysis.
