# Supplementary material for: How Does Vaccination against SARS-CoV-2 Affect Hospitalized Patients with COVID-19?
Source: J Clin Med. 2022 Jul 5;11(13):3905. doi: 10.3390/jcm11133905 (PMC9267443; doi:10.3390/jcm11133905)

SUPPLEMENTARY TABLE S1. Charlson Index

Comorbidities - Group	CIE-10 – Search Patterns	Weight
Myocardial infarction	#I21, #I22, #I25.2#	1
Heart failure	#I09.81#, #I11.0#, #I13.0#, #I13.2#, #I25.5#, #I42.0#,#I42.5#, #I42.6#, #I42.7#, #I42.8#, #I42.9#, #I43#, #I50,#P29.0#	1
Peripheral vascular disease	#I70, #I71, #I73.1#, #I73.8, #I73.9#, #I77.1#, #I79.0#, #K55.1#, #K55.8#, #K55.9#, #Z95.8, #Z95.9#	1
Cerebrovascular disease	#G45, #G46, #I6	1
Dementia	#F01, #F02, #F03, #G30, #G31.1#	1
Chronic lung disease	#I27.8, #I27.9#, #J4, #J60#, #J61#, #J62, #J63, #J64#, #J65#, #J66, #J67, #J68.4#, #J70.1#, #J70.3#	1
Rheumatic diseases	#M05, #M06, #M31.5#, #M32, #M33, #M34, #M35.1#, #M35.3#, #M36.0#	1
Peptic ulcer disease	#K25, #K26, #K27, #K28	1
Mild liver disease	#B18, #K70.0#, #K70.1, #K70.2#, #K70.3, #K70.9#, #K71.3#,#K76.3#, #K76.4#, #K76.8, #K76.9#, #Z94.4#	1
Diabetes without chronic complications	#E10.1, #E10.6, #E10.8#, #E10.9#, #E11.0, #E11.6, #E11.8#, #E11.9#, #E13.0, #E13.1, #E13.6, #E13.8#, #E13.9#	1
Diabetes with chronic complications	#E10.2, #E10.3, #E10.4, #E10.5, #E11.2, #E11.3, #E11.4, #E11.5, #E13.2, #E13.3, #E13.4, #E13.5	2
Hemiplegia or paraplegia	#G04.1#, #G11.4#, #G80.1#, #G80.2#, #G81, #G82.2, #G83.0#, #G83.1, #G83.2, #G83.3, #G83.4#, #G83.9#	2
Renal disease	#I12.0#, #I13.1, #N03.2#, #N03.3#, #N03.4#, #N03.5#,#N03.6#, #N03.7#, #N05.2#, #N05.3#, #N05.4#, #N05.5#,#N05.6#, #N05.7#, #N18, #N19, #N25.0#, #Z49, #Z94.0#,#Z99.2#	2
Malignant neoplasms, including lymphoma and leukemia, and excluding those of the skin	#C0, #C1, #C2, #C3, #C40, #C41, #C43, #C45, #C46, #C47, #C48, #C49, #C5, #C6, #C70, #C71, #C72, #C73#, #C74, #C75, #C7A, #C7B, #C76, #C81, #C82, #C83, #C84, #C85, #C88, #C9	2
Moderate or severe liver disease	#I85.0, #I86.4#, #K70.4, #K71.1, #K72.1, #K72.9, #K76.5#,#K76.6#, #K76.7#	3
Solid metastatic tumor	#C77, #C78, #C79, #C80	6
AIDS and HIV infection	#B20#	6

SUPPLEMENTARY FIGURE S1. Temporal distribution of receipt of the first dose of the vaccine according to the age group to which they belonged. Only fully vaccinated patients (n=78) are represented.

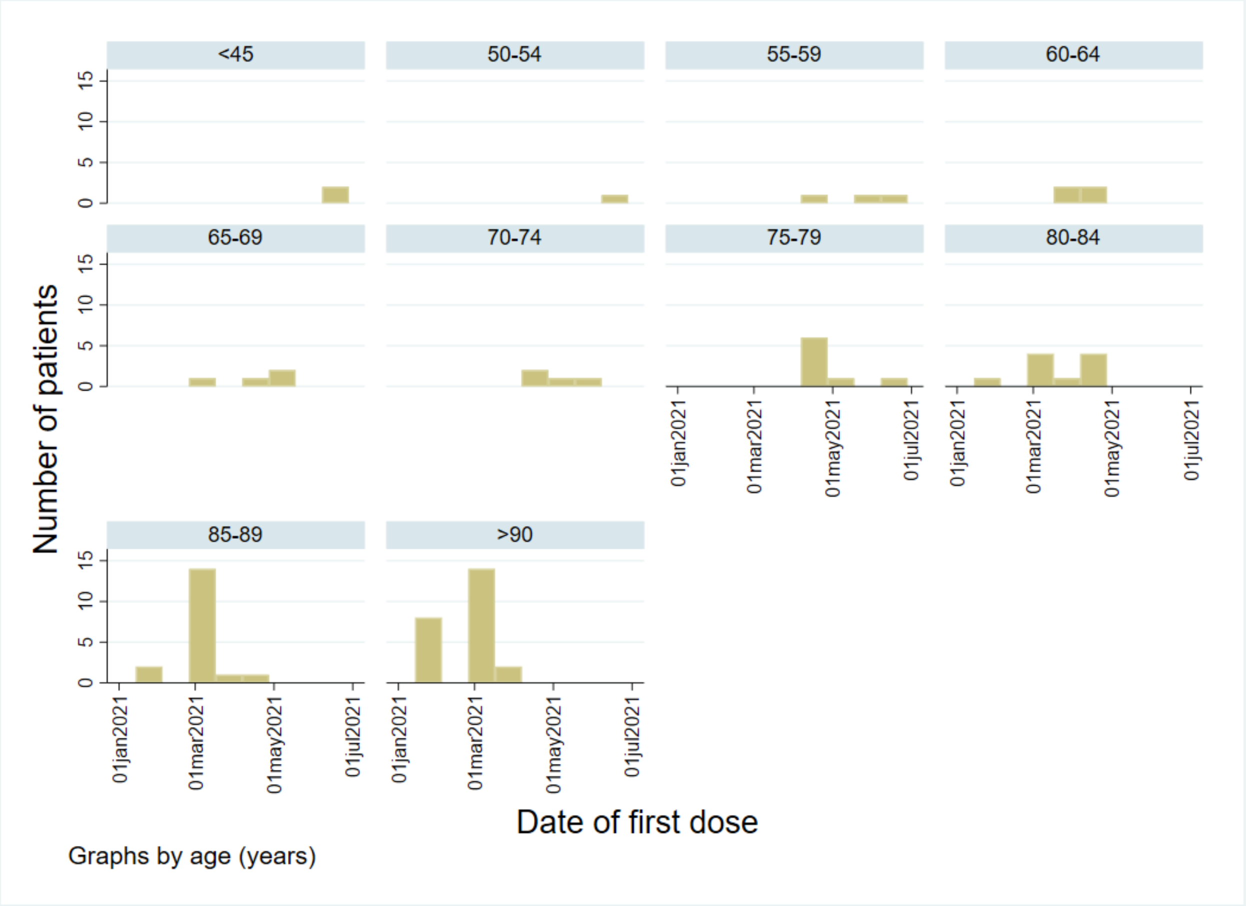

*

SUPPLEMENTARY FIGURE S2. Calibration plot of mortality model

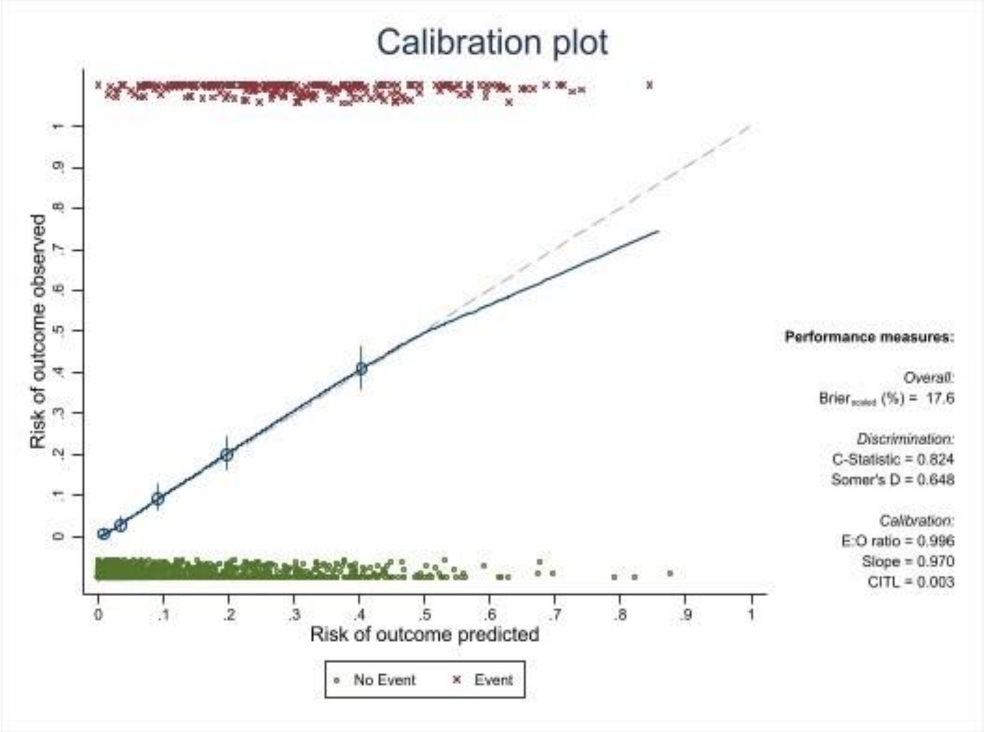

SUPPLEMENTARY FIGURE S3. Linear regression model residuals analysis

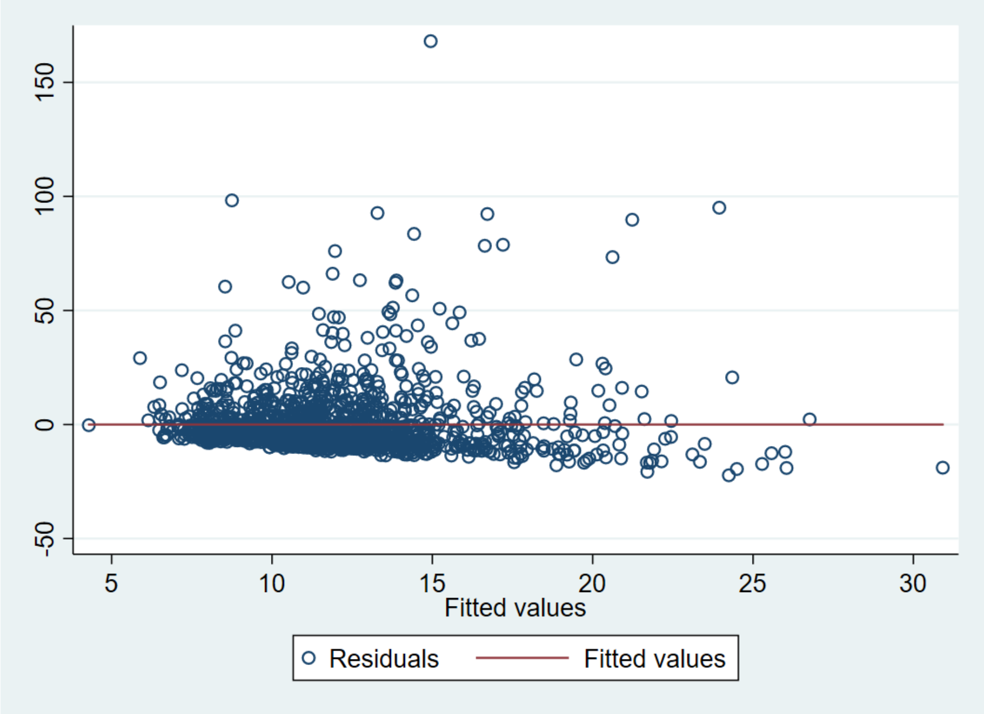

Supplement: Supplementary file 1 [file jcm-11-03905-s001.zip › jcm-1779624-supplementary.pdf]
